# Toward a Unified View of Cognitive and Biochemical Activity: Meditation and Linguistic Self-Reconstructing May Lead to Inflammation and Oxidative Stress Improvement

**DOI:** 10.3390/e22080818

**Published:** 2020-07-27

**Authors:** Carlo Dal Lin, Laura Brugnolo, Mariela Marinova, Mario Plebani, Sabino Iliceto, Francesco Tona, Giuseppe Vitiello

**Affiliations:** 1 Department of Cardiac, Thoracic and Vascular Sciences, Padua University Medical School, Via Giustiniani 2, 35100 Padua, Italy; sabino.iliceto@unipd.it (S.I.); francesco.tona@unipd.it (F.T.); 2 Department of Laboratory Medicine, Padua University Medical School, Via Giustiniani 2, 35100 Padua, Italy; sid_11@hotmail.it (L.B.); mariela.marinova@aopd.veneto.it (M.M.); mario.plebani@unipd.it (M.P.); 3 Department of Physics “E.R. Caianiello”, Salerno University, Via Giovanni Paolo II, 132, 84084 Fisciano (Salerno), Italy

**Keywords:** stress, cognition, relaxation response, meditation, dissipative quantum model of brain, language, Rational–Emotional–Education

## Abstract

Stress appears to be the basis of many diseases, especially myocardial infarction. Events are not objectively “stressful” but what is central is how the individual structures the experience he is facing: the thoughts he produces about an event put him under stress. This cognitive process could be revealed by language (words and structure). We followed 90 patients with ischemic heart disease and 30 healthy volunteers, after having taught them the Relaxation Response (RR) as part of a 4-day Rational–Emotional–Education intervention. We analyzed with the Linguistic Inquiry and Word Count software the words that the subjects used across the study following the progression of blood galectin-3 (inflammation marker) and malondialdehyde (oxidative stress marker). During the follow-up, we confirmed an acute and chronic decrease in the markers of inflammation and oxidative stress already highlighted in our previous studies together with a significant change in the use of language by the subjects of the RR groups. Our results and the precise design of our study would seem to suggest the existence of an intimate relationship and regulatory action by cognitive processes (recognizable by the type of language used) on some molecular processes in the human body.

## 1. Introduction

Stress appears to be the basis of many diseases [1]. Although a stress condition is undoubtedly linked to triggering events, the link between “stress” and “events” does not seem to be causal, nor necessary, nor sufficient. Different people, facing the same situation, respond differently on a psycho-emotional and behavioral level. But even one single person, in the course of a lifetime, can face the same situation or experience the same problem differently.

In the same way, the stress reaction is independent of the presence of pathologic psychological comorbidities [2].

Central is how the individual, consciously or not, structures the experience he is facing [3]: the thoughts he produces about that event put him under stress [4,5]. The subject reacts to the perceptual inputs through the brain’s action-perception cycle and his emotions and behaviors are generated [6]. Although this dynamical integration process is still not fully understood and under study, it appears that language plays a relevant role in it [7,8,9,10,11,12,13,14,15,16,17,18,19].

The individual’s stereotyped methods of response to the environment that surrounds him fall within the concept of “personality” [20,21], namely the subject is in a highly nonlinear interaction with his environment. It has been amply demonstrated that there are personalities more likely to develop psychological distress [4] and, consequently, stress-related pathologies [20,21,22].

As personality seems to be in strict relation with language [23], it is possible to identify several personality traits through the language used by a person [24]. Words are indicators of how the subject is structuring his reality, the flow of his attention, his attitudes, his limiting beliefs, etc. [25] (see below in the Discussion section). A ready-to-use tool to start to analyze these aspects is provided by appropriate software such as the Language Inquiry Word Count (LIWC) [24].

Recently, we followed for one year some patients with ischemic heart disease and some healthy volunteers, after having taught them the Relaxation Response (RR) as part of a 4-day Rational–Emotional–Education intervention and we documented a significant decrease in the perceived degree of stress combined with a clinically favorable variation of different neuro-endocrine-immune markers of inflammation, oxidative stress [26] and circulating microRNAs [27].

Given the introductory premises, we asked ourselves if the progressive well-being perceived by the subjects enrolled could be linked to a progressive restoration of their personality and their language.

To answer this question, we analyzed with the Linguistic Inquiry and Word Count (LIWC) software (http://www.liwc.net) the words that the subjects used across the study, during the first educational days, in the two follow-up meetings and their emails. To confirm our observations from a biochemical point of view, we followed the progression of galectin-3 (GAL-3) as a marker of inflammation and malondialdehyde (MDA) concerning oxidative stress.

## 2. Materials and Methods

### 2.1. Biological Samples

We analyzed the serum samples of 120 subjects following an approved protocol (protocol number 3487/AO/15—13/7/2015) [26]. Briefly, we enrolled 90 consecutive patients after myocardial infarction and 30 healthy controls. Thirty patients were taught to meditate, 30 to appreciate music, and 30 did not carry out any intervention and served as controls. To rule out that the disease state could interfere with the relaxation effect, we enrolled 30 healthy volunteers (15 were trained to meditate and 15 had music appreciation). The practices of meditation and music appreciation can produce the so-called Relaxation Response (RR) in the same way [28]. The details of the RR techniques that we used and the description of their pathophysiological mechanism is described in our previous works [26,27].

**Figure 1:**
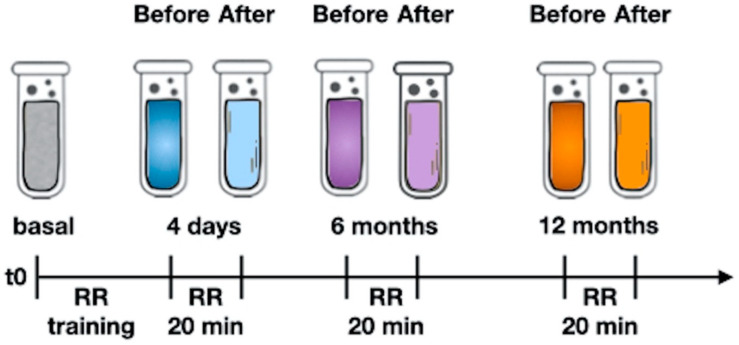
The study design. Explanation in the text. RR: Relaxation Response. RR 20 min: after 4 days of training, each subject relaxes through meditation or music appreciation for 20 min. A blood sample is taken immediately before and immediately after. The acute variation of the studied parameters can be attributed to the practice of relaxation according to the used methods because the precise timing of blood sampling (before and immediately after the end of the session) prevents any other influences. All groups were subjected to the same environmental conditions: in particular, also the control patients were taken in our classroom for 20 min and were not subjected to any intervention. We simply asked them to relax and most of them sat down with eyes closed. For more details please see our previous works [26,27].

After the initial four-days-training, after 6 and 12 months of RR practice, we collected a blood sample immediately before and after the relaxation session (according to the scheme reported in Figure 1) to describe any variation of GAL-3 and MDA, the two markers that moved with greater sensitivity in our previous work [26].

**Figure 2:**
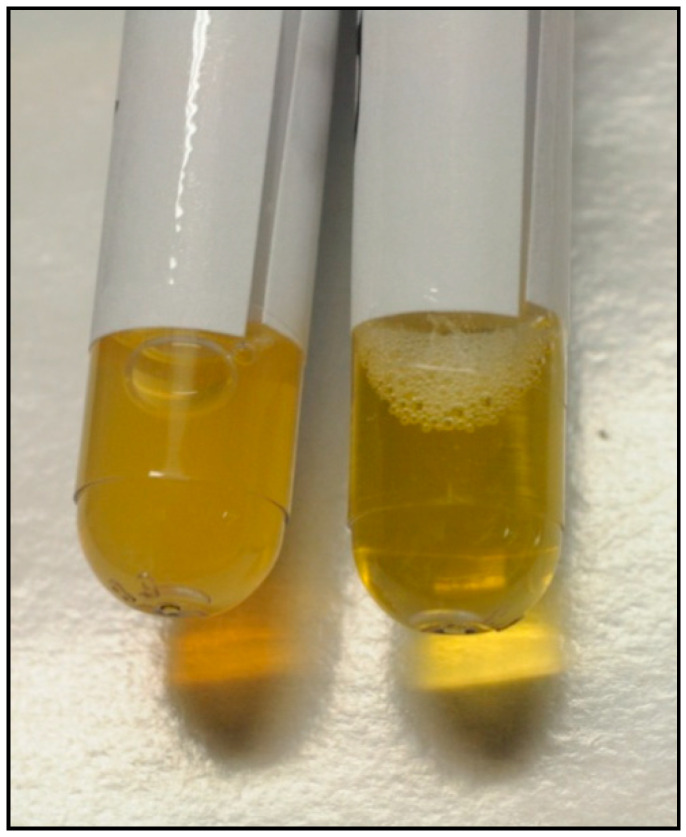
Variation of the physical characteristics of the plasma of the same patient during 20 min of meditation. On the left: the blood sample (after 4 min of centrifugation at 5000 rpm) before meditation is opalescent. On the right, the blood sample immediately after meditation is clearer. The patient was fasting for more than 5 h before meditating.

Clear variation of the physical characteristics of the serum samples (Figure 2), was observed.

According to Benson’s researches [18,29,30] and our previous study [26,27], there are no significant differences between relaxation techniques. Therefore, we merged into a single “intervention” group (called “RELAXATION RESPONSE”) all patients treated with meditation and music and into a single “intervention healthy controls” group (called “RELAXATION RESPONSE HEALTHY CONTROLS”) all healthy subjects. Finally, the patients that did not carry out any intervention constituted the “CONTROLS” group.

We emphasize that in our work we observed the RR using two conditioning techniques, meditation, and music, which have to be considered as two ways leading to the same relaxation effect [18]. Therefore, even from a strictly methodological point of view, we used a unique technique—precisely the RR—from which also the need to unite in a single “intervention group” the treated subjects.

Indeed, all subjects enrolled in the study have continued the practice at home, twice a day, as they taught. During the follow-up period, each subject reported having pleasantly performed more than 80% of the meditation or music listening sessions.

### 2.2. Language Evaluation

During the study, we recorded all the words spoken by the participants in the training meetings of the first 4 days and during the follow-up meetings at 6 and 12 months. We also analyzed the emails that periodically sent us during the study. In particular, each participant sent an email about 2 months and 4 months from the start of the study telling about their experience. Then, after the blood sample control at 6 months, another email at 8 and 10 months was sent before the final appointment at 12 months. All the texts have been analyzed through the LIWC software (http://www.liwc.net).

We divided the texts collected in the first 6 months of the study—initial training meetings and the two e-mails at 2 and 4 months—(the analyzed data referring to the first 6 months of the study were called “START”), from those collected in the final 6 months (6 and 12 months follow-up meetings and the two e-mails at 8 and 10 months—the analyzed data referring to the last 6 months of the study were called “END”). Then, we compared the means and standard deviations of the items provided by the software in the same subjects and among the RELAXATION RESPONSE group versus the CONTROLS and between the RELAXATION RESPONSE group and RELAXATION RESPONSE HEALTHY CONTROLS.

The items provided by the software are quite intuitive, for an explanation of them please see: https://repositories.lib.utexas.edu/bitstream/handle/2152/31333/LIWC2015_LanguageManual.pdf.

### 2.3. Statistical Analysis

GAL-3 and MDA levels are expressed as median and interquartile range. LIWC items are expressed as mean ± standard deviation. For biochemical markers, the comparison between the pre-post intervention changes was performed using Wilcoxon test. The comparison between groups was performed through the Mann–Whitney test. T-tests for dependent and independent samples was used to analyze language items. The distribution of the individual variables was assessed by the Shapiro–Wilk test. An initial comparison between groups was performed by means of Kruskal–Wallis test for independent samples or by Friedman test for paired data. Statistical significance was assumed if the null hypothesis could be rejected at *p* < 0.05. The statistical analysis was performed using software SPSS version 22.0 (Chicago, SPSS, Inc., Chicago, IL, USA). All conditions that could have affected the improvement of the serum molecules have not changed and are comparable in the different groups at baseline, after 6 months and after 12 months (same physical rehabilitation and nutritional support, same therapy, same time of follow-up and same environmental conditions at the time of sampling).

#### Clarification on the Concept of Statistical Significance in Biology/Medicine

The P-value indicates the probability that two apparently different groups of data actually come (or not) from the same population and does not indicate whether the hypotheses under study is true or false [31].

Therefore, when the reader will observe in our graphs and tables that two distributions of data “differ in a statistically significant way, *p* < 0.05” it means that the analyzed data could theoretically belong to two different populations.

Statistical methods were applied in biomedical research to allow us to quantify uncertainty; they were not developed as a decision tool or as an instrument of dogmatic truth. The use of a *p*-value of 0.05 as a threshold for declaring statistical significance is just a simple clinical/biological convention, still under debate [32].

## 3. Results

### 3.1. Biochemical Markers

**Figure 3:**
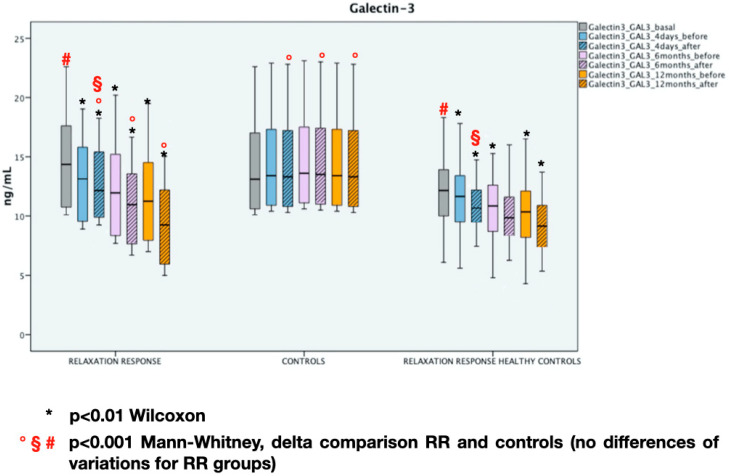
Galectin-3 (GAL-3) at different time points. Median and interquartile range, statistical analysis.

**Figure 4:**
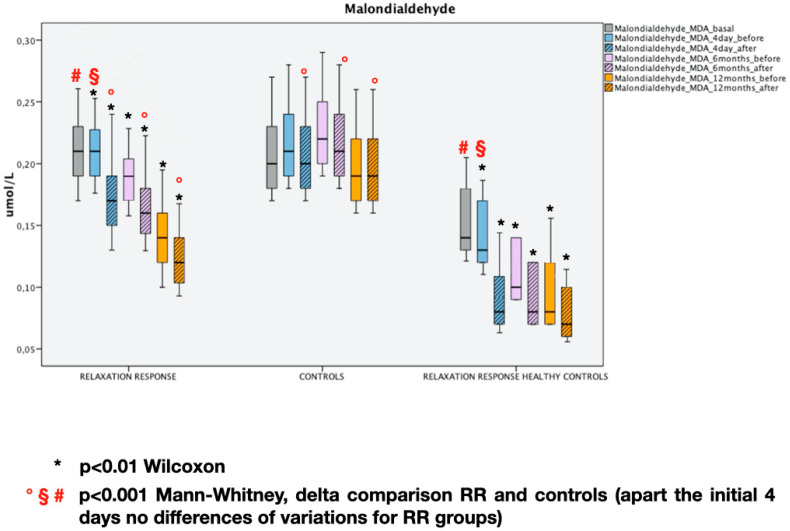
Malondialdehyde (MDA) at different time points. Median and interquartile range, statistical analysis.

The results along with the statistical analysis of GAL-3 and MDA are reported in Figure 3 and Figure 4. The RR results in a significative decreasing of GAL-3 and MDA at every time point (*p* < 0.01 Wilcoxon test at every time point) while in the Controls do not vary (*p* > 0.05 at every timepoint). On the one hand, it is possible to notice that healthy controls have lower starting levels of both inflammation and oxidative stress markers than RR group (*p* < 0.001 Mann–Whitney test—basal), on the other hand, the behavior of the same markers is similar in individuals subjected to RR (*p* > 0.05 Mann–Whitney test at every time point).

### 3.2. Language Results

The results along with the statistical analysis of linguistic items are reported in Table A1 and Table A2 in the Appendix A. To make the results easier to follow, we summarized in Table 1 the trend of each item during the study in the three groups. The up arrow indicates a statistically significant increase during follow-up, the down arrow the opposite, the dash indicates no significant changes.

## 4. Discussion

The biological world operates on a multitude of scales from atoms to molecules, from cells to macro-organisms and ecosystems. All these levels are connected by a continuous flow of information made of electro-chemicals mediators, by physical forces and in-phase correlations and constitute a global system characterized by specific macroscopic behaviors.

Stress is the basis of many diseases, particularly cardiovascular ones [33]. It is accompanied by the perception of negative emotions such as depression, anxiety, anger, fear, or panic [34]. Each of these emotions can have dramatic consequences on the cardiovascular level [35].

But emotional expression can be effectively regulated by voluntary cognitive control processes [36].

During the 4 days of subject RR training, we taught how to try to rework their negative emotions to mitigate their biological effect. During the reworking process, we know that the activity of the prefrontal cortex increases with the reduction of the amygdala function [37,38], whose hyperactivation seems to promote inflammation and damage the heart [39].

Therefore, during the two daily sessions of 20 min of RR, brain attention network activates while the subject interrupts the flow of ordinary thoughts concentrating on the object of meditation (in this case a word sound or music) [40]; the parasympathetic tone prevails, and a series of neuroendocrine changes occur [26,27] leading to an acute attenuation of oxidative stress and inflammation (macroscopically, the physical appearance of the blood plasma also varies—Figure 2. We comment more on this point in the following).

We have to consider that Gal-3, besides playing an important regulatory role in inflammation, modulates basic cellular functions such as cell–cell and cell–matrix interactions, growth, proliferation and differentiation and it is involved in the pathogenesis of many relevant human diseases, including cardiovascular disorders and cancer [41]. The MDA molecule is able to interact with nucleic acid bases to form several different adducts able to induce sequence-dependent frameshift mutations and base-pair substitutions that may lead to cancer [42]. Nonetheless, MDA toxicity is directed also towards cardiovascular stability, modifying lipoproteins and impairing their interaction with macrophages, a key step of atherogenicity [43].

As briefly mentioned in the Introduction, language has an important role in the construction and development of some of the personality traits. Personality is much affected by the surroundings, since the subject can be described as a system permanently open to its environment. In its functional activity, the brain finds in the world the source of its energy needs and the sink where its energy waste goes. The persistent search of the harmonious balance of the outgoing and ingoing fluxes of energy and information between the subject and its world is at the core of the action-perception cycle characterizing brain activity [44,45,46,47,48,49,50,51].

Since the balance arises from the outgoing (forward in time) and ingoing (time-reversed) fluxes, with reference, e.g., to the subject, the world appears then to be the *Double* of the subject, his image in the mirror of time [44,45,46]. The openness of the subject to the environment finds then its realization in the permanent *dialogue* between the self and its Double. Such a “talking” between the two (sometimes felt as a “talking inside” since the Double is indeed the subject’s image) is therefore crucial in the continuous *trade* of the self with the world in the search of the best *to-be-in-the-world*. Any event or situation judged as “stressful” then introduces more or less severe perturbations unbalancing the energy flow between the self and the Double and it is not surprising that these unbalancing perturbations affect the *words* of the *language* through which the dialogue between the self and its Double is realized. We see that all of this is a strict consequence of the open (i.e., dissipative) character of the brain dynamics, which supports the thesis of the immanence of some of the linguistic structures to the brain physical dynamical processes (by resorting to results in Chomsky’s Minimalist Program (MP) [12,13], it has been shown [9,10,11] that specific linguistic features admit a representation in terms of the algebraic formalism used in the formulation of the dissipative quantum model of the brain [44,45,46]).

One important point which clarifies the relevance of the RR techniques in recovering from stress is that there is always a content of *attention* in the perception, so that among the many perceptual stimuli arriving from the world, the subject selects, focus only on the ones which he associates to a “value”, worth to expend energy on it. There is then always an *intention* content in the action aimed at the best to-be-in-the-world [44,45,46,47,48,49,50,51]. Out of this, the formation of *meanings* arises. Meanings emerge from the dynamical correlations with the world constructed during the perceptual history of the subject. *Intentionality* and *meaningfulness* are the basic ingredients of the subject *identity*, which manifests in its personality.

The harmonious to-be-in-the-world mentioned above, which according to Desideri constitutes the subject’s aesthetic experience [52,53], is damaged by events judged as “stressful”. The subject is then guided through the RR therapy to repair and recover a peaceful talking with its Double. It has to be observed that in the dissipative quantum model the act of consciousness is postulated to arise exactly in the dialogue with the Double [44,45,46], and thus it manifests itself in the words between the two. We also remark that there is entanglement between the self and its Double [46], so that they cannot be separated, they are always “an undivided two”, in phase resonating, so that words among them are not really messenger carrying information, they express the meaningfulness of their correlation. In this sense, we were saying in the Introduction that words are indicators of how the subject is structuring his reality, his vision of the world. From such a standpoint the use of the LIWC software in our analysis has to be regarded as the first, readily available tool to look at changes in the statistics of the elementary language components. Nevertheless, it is interesting to see that the use of the pronouns, the specificity of the emotions, the tenses of the verbs, are actually fitting with the remarks and considerations made above in our discussion. In fact, the rational-emotional education and the RR processes have allowed, over time, to restructure the personality of the subjects [54].

The patients we followed seem to be more aware of the experiences they lived, as witnessed by the change in their language of some syntactic structures. According to Yarkoni [24] and Pennebaker’s works [55], the changing of personal pronouns use that we documented, may reveal increased attention to social dynamics along with greater awareness in self boundaries; words linked to positive emotions together with cognitive verbs have also increased; verbal tenses are more declined in the present, in an active and not passive form, with an increase of verbs indicating “choice” and “will”, rather than “duty”; there has been also an increase in the use of definite articles and, in general, a lesser description of life events and a greater attention to the personal experience. Words and phrases have become shorter as if to underline more immediate experiences and greater presence of the subject in his reality. All of it pointing to the restoring of the more harmonious dialogue with the Double.

Thus, in the long term, we speculate that the combination of daily relaxation and, especially, the progressive psycho-emotional re-elaboration may have contributed to attenuate the psychological triggers of inflammation and oxidative stress unlike controls (with equal diet, physical activity, and medical therapy).

Emotions, although triggered by events, express the meaningfulness of our relationship with the world (how the dialogue with our Double develops). Our data seem to suggest that if the structure and sequence of thoughts do not change, emotions and behaviors do not change, even if external circumstances can apparently improve.

Moreover, our data regarding control patients may indicate that when a man constructs and follows thoughts of judgment, disapproval, separation, or condemnation, he automatically prepares himself to feel emotions coherent with those thoughts (such as annoyance, irritation, anger, malaise, suffering, sadness, etc.). Remaining in this structure of thought means remaining in these emotions letting the relative molecular mediators that regulate them flow inside the body. Even if the mechanisms by which this happens are still unknown, these psychological operative modalities can be reworked and replaced with others more adaptive and able to promote resilience, well-being and serenity.

As discussed elsewhere [9,10,11], the conceptual linguistic content (meaning) then emerges in a dynamical process going from the syntactic level of lexical elements to the semantic level of the “manifold of concepts” (called in the MP, the set of Logical Forms (LF) [56]). The mathematical formalism describing such a process has been shown [9,10,11] to be isomorph to the one describing in condensed matter physics the formation of coherent states starting from elementary components. In the jargon of the MP, this is expressed by saying that the Narrow Syntax makes contact at the interfaces with the conceptual interpretative (CI) system. The sensory-motor system (SM) is then involved through the dynamics of the cortex by means of the action–perception cycle [44,45,46,47,48,49,50,51]. The result is that the externalization of language expressions becomes possible in a way to manifest the meaningfulness of the subject’s perceptual experiences.

The mentioned coherence of the (brain) states refers to the collective dynamics of the synchronous amplitude modulated (and phase-modulated) oscillations of neuronal assemblies (which are observable by means of Electroencephalogram (EEG), functional Magnetic Resonance Imaging (fMRI), and other imaging techniques). The collective dynamics is due to long-range neuronal correlations whose dynamical generation is triggered by the perceptual inputs (and maybe heart inputs [57,58]). On the linguistic side, the picture which emerges is that coherent waves spanning lexical components are at the basis of the concept’s generation [9,10,11]. Concepts are thus not specifically associated with individual lexical components. They are instead formally described as collective modes corresponding to the ordering of lexical elements.

Coherence also ensures the stability of the semantic content and corresponds to the minimization of free energy [9,10,11]. The language changes detected by us by means of the LIWC software analysis thus go beyond mere changes in the statistics of the elementary language components of the patient under study. They actually signal changes in the subject’s semantic landscape, namely its rearrangement into a new scenario corresponding to a different minimum free energy state (one can show indeed that the “concept space” includes subspaces, each one endowed with its minimum free energy state [9,10,11]).

On more remark concerns the nonlinearity of the interaction of the subject with its environment. The mathematical description in such a circumstance requires to consider both, the system of interest (the subject) and also its environment, both of which, as said above, must be considered in their persistent interaction. Formally this amounts to the doubling of the degrees of freedom, say A → A × A, where A in the first position denotes the system degrees of freedom (more precisely the neuronal degrees of freedom in the dissipative quantum model of the brain) and the “doubled” A, which we denote as Ã, the ones of the environment (the Double). One can show that the entanglement mentioned above is the one between the A and Ã modes [46,47,48,49,50,51]. It implies that observables depending on A are actually determined by Ã (and vice-versa), so that Ã modes act as the “address”, or the “dynamical reference” of the A modes (and vice-versa). From the linguistic standpoint, it is known that lexical elements may have “copies” which are “seen” at the conceptual interpretative (CI) system level, but remain silent (are not pronounced) at the sensory-motor (SM) system (e.g., in the sentence *Which paintings did you see [paintings]?* or *John saw a man at the corner of the street, and Marc did_ _ _too.*) (sometimes a whole syntactic component is omitted and a “syntax of silence” seems to exist [59]). Here, we did not go further in the analysis of these linguistics aspects. We only remarked that including in the formalism the copies of lexical elements allows the possibility of dynamical matching between the two and a “truth evaluation function”, or logical self-consistency, appears to be built-in in languages [9,10,11]. One of the effects of the RR therapy is then the reinforcement of *truthfulness* and *realism* feelings in the treated patients.

In summary, the strict interplay between language and thoughts [60,61,62], seems to be a tool of primary importance both at a diagnostic and therapeutic level, since, on the one hand, it seems to reveal the psycho-emotional positions of the person and, on the other, it allows them to be modified if necessary.

Recent evidence suggests that language in terms of “sound” can have a direct effect on cell biological structures [63] and is able to create coherent orders within biological fluids such as plasma [64].

Let us close with a final comment on this specific point. As already discussed in previous works [26,27,64] we have observed that the serum pH of subjects who undergo a Relaxation Response practice (Figure 2) could significantly increase while electric conductivity seems to decrease. According to the many-body physics modeling [64,65,66,67], the efficient metabolic activity in healthy biological systems is favored by coherent dynamics at intra- and inter-cellular levels. The system energetic feeding by ATP hydrolysis or by other sources is used for the generation of organizational activity, namely the formation of long-range coherent correlation modes among the electric dipoles characterizing macromolecules and the molecules of the water bath in which they are embedded. These correlation modes are responsible of the non-vanishing polarization density *P*(*x*,*t*) (the electrets) [66,67]. Chemical, oxidative stress or else functional, mechanical or electromagnetic agents may affect negatively the coherent molecular ordering.

A common laboratory observation [68,69,70,71] is that water molecular dipoles organize themselves in coherently ordered strata adjacent to hydrophilic polymers (for example nafion), extending for a thickness of a hundred of microns. The coherent ordering of these strata expels present particles and is impenetrable by them or other impurities. The strata are called EZ (exclusion zones). Their polarization is such that charges opposite to the ones of the material surface (negative in the case of nafion) are pushed out of the strata. In the direction orthogonal to the surface, the pH shows then a gradient: in the nafion case, pH is lowering moving far away from the surface. Moreover, observations show that in the EZ region it is energetically advantageous the splitting of water molecules into OH− and H+ [68,69,70,71], which of course affects the electrical conductivity [71]. Measurements also show that for water in the presence of nafion [72] higher pH corresponds to lower electric conductivity (and vice-versa).

These experimental observations suggest that similar behavior of pH, electrical conductivity and charge distribution may be also induced in biological fluids in the presence of surfaces such as those of cell membranes, of veins and arteries; a hypothesis which is indeed consistent with our measurements in the Relaxation Response practice [63]. The higher transparency of the plasma in Figure 2 in the post-RR practice case is also consistent with the higher purity of the fluid and higher coherence in its constituent organization, namely with a much lower number of photon scattering processes with “non-correlated” scattering centres (molecules), i.e., lower energy losses by diffusion of photons (they “see” the plasma as a global system, not as a collection of quasi-independent molecular components to scatter with and losing energy (lower transparency)).

The coherence in the plasma microscopic dynamics is, of course, consistent with the general coherent dynamics referred to in our discussion above on brain, RR practice, and language functional features.

As a perspective conclusion, the correspondences between language, stress, inflammation, oxidative stress, and somatization that we have described in this work, although still speculative in many respects, appear to be pointing to a unifying view of mental activity and biochemical activity [49,63,64,65]. Much work along such research lines has still to be done.

## Figures and Tables

**Table 1:** Trend of the language analysis variations according to the Language Inquiry Word Count (LIWC) items between RELAXATION RESPONSE group, CONTROLS, and RELAXATION RESPONSE HEALTHY CONTROLS.

ITEM	RELAXATION RESPONSE	CONTROLS	RELAXATION RESPONSE HEALTHY CONTROLS
*Word count, Words per sentence, Sentences ending with ? % words longer than 6 letters*	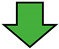	-	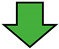
*Total pronouns*	-	-	-
*1st person singular*	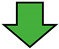	-	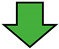
*1st person plural*	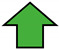	-	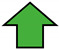
*Total first person*	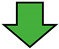	-	-
*Total second person*	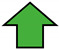	-	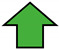
*Total third person*	-	-	-
*Negation*	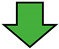	-	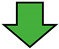
*Assents*	-	-	-
*Articles*	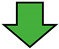	-	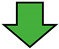
*Prepositions*	-	-	-
*Numbers*	-	-	-
*Affective/emotional processes, Positive emotions, Optimism and energy*	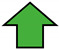	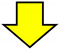	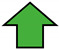
*Negative emotions, Anxiety or fear, Anger, Sadness or depression*	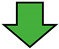	-	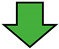
*Cognitive processes, Causation*	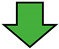	-	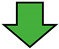
*Insight*	-	-	-
*Discrepancy, Inhibition*	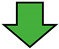	-	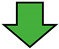
*Tentative, Certainty*	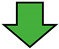	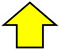	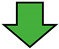
*Sensory and perceptual processes, Social processes*	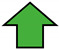	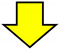	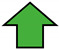
*Past tense verb, Future tense verb*	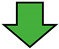	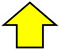	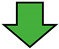
*Present tense verb*	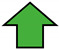	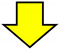	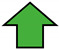
*Inclusive*	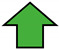	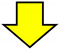	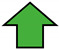
*Exclusive*	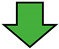	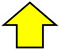	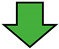
*Motion, Leisure activity*	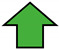	-	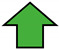
*Money and financial issues*	-	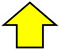	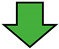
*Body symptoms*	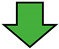	-	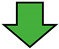
*Body functions*	-	-	-

## Appendix A

**Table A1:** Comparison of the language analysis according to the LIWC items between RELAXATION RESPONSE group and CONTROLS. The *p* describing the Intra-group comparison is under, the *p* describing the inter-group comparison is on the right.

		RELAXATION RESPONSE	CONTROLS	*p*
*Word count*	START	344.69 ± 51.44	339.32 ± 47.94	>0.05
	END	244.69 ± 34.11	329.32 ± 51.71	<0.001
	*p*	<0.01	>0.05	
*Words per sentence*	START	22.81 ± 5.71	23.01 ± 7.39	>0.05
	END	13.54 ± 3.62	24.87 ± 6.89	<0.05
	*p*	<0.01	>0.05	
*Sentences ending with ?*	START	1.82 ± 1.07	1.95 ± 0.86	>0.05
	END	0.69 ± 0.72	2.07 ± 1.1	<0.001
	*p*	<0.01	>0.05	
*% words longer than 6 letters*	START	18.6 ± 3.62	17.67 ± 2.11	>0.05
	END	10.6 ± 1.74	17.9 ± 3.71	<0.01
	*p*	<0.001	>0.05	
*Total pronouns*	START	70.15 ± 14.04	64.72 ± 13.53	>0.05
	END	64.82 ± 14.69	68.49 ± 13.71	<0.05
	*p*	>0.05	>0.05	
*1st person singular*	START	10.15 ± 2.83	10.01 ± 2.82	>0.05
	END	5.15 ± 1.97	9.87 ± 2.81	<0.01
	*p*	<0.001	>0.05	
*1st person plural*	START	6.88 ± 3.54	7.15 ± 2.95	>0.05
	END	9.88 ± 2.44	6.75 ± 3.13	<0.05
	*p*	<0.05	>0.05	
*Total first person*	START	17.02 ± 3.62	17.16 ± 4.66	>0.05
	END	14.03 ± 2.51	16.62 ± 4.64	>0.05
	*p*	<0.05	>0.05	
*Total second person*	START	10.6 ± 2.49	10.47 ± 2.47	>0.05
	END	13.6 ± 1.18	10.54 ± 2.69	<0.05
	*p*	<0.01	>0.05	
*Total third person*	START	8.07 ± 1.97	7.57 ± 1.68	>0.05
	END	8.04 ± 1.84	8.02 ± 1.85	>0.05
	*p*	>0.05	>0.05	
*Negation*	START	16.08 ± 3.8	16.58 ± 2.81	>0.05
	END	11.08 ± 2.6	16.04 ± 2.58	<0.001
	*p*	<0.001	>0.05	
*Assents*	START	6.05 ± 1.95	6.71 ± 1.79	>0.05
	END	5.97 ± 1.89	6.62 ± 2.18	>0.05
	*p*	>0.05	>0.05	
*Articles*	START	11.74 ± 3.16	11.36 ± 3.23	>0.05
	END	8.74 ± 3.25	11.27 ± 2.76	<0.01
	*p*	<0.01	>0.05	
*Prepositions*	START	18.93 ± 4.62	18.16 ± 4.52	>0.05
	END	20.93 ± 4.12	19.07 ± 4.61	>0.05
	*p*	>0.05	>0.05	
*Numbers*	START	11.87 ± 2.31	12.06 ± 2.25	>0.05
	END	12.26 ± 2.34	11.76 ± 2.42	>0.05
	*p*	>0.05	>0.05	
*Affective/emotional processes*	START	24.07 ± 6.79	24.86 ± 7.28	>0.05
	END	31.07 ± 7.27	19.86 ± 7.35	<0.001
	*p*	<0.01	<0.01	
*Positive emotions*	START	17.61 ± 4.57	17.86 ± 4.87	>0.05
	END	22.61 ± 3.67	16.94 ± 3.88	<0.001
	*p*	<0.01	>0.05	
*Optimism and energy*	START	27.55 ± 5.19	27.56 ± 5.55	>0.05
	END	32.55 ± 4.68	24.56 ± 6.45	<0.05
	*p*	<0.05	>0.05	
*Negative emotions*	START	39.83 ± 9.42	41.32 ± 6.58	>0.05
	END	31.83 ± 7.31	43.32 ± 7.77	<0.01
	*p*	<0.01	>0.05	
*Anxiety or fear*	START	52.57 ± 11.39	50.78 ± 11.53	>0.05
	END	37.57 ± 9.29	52.78 ± 10.87	<0.001
	*p*	<0.001	>0.05	
*Anger*	START	25.66 ± 6.44	26.04 ± 5.93	>0.05
	END	14.66 ± 5.25	23.04 ± 6.63	<0.01
	*p*	<0.001	>0.05	
*Sadness or depression*	START	44.69 ± 9.26	45.06 ± 9.36	>0.05
	END	29.69 ± 7.76	41.06 ± 10.46	<0.001
	*p*	<0.001	>0.05	
*Cognitive processes*	START	31.42 ± 9.44	35 ± 9.89	>0.05
	END	24.42 ± 7.41	38 ± 7.19	<0.001
	*p*	<0.01	>0.05	
*Causation*	START	16.47 ± 3.67	16.52 ± 3.76	>0.05
	END	12.47 ± 2.17	22.52 ± 4.76	<0.01
	*p*	<0.01	>0.05	
*Insight*	START	9.64 ± 3.63	8.54 ± 4.16	>0.05
	END	9.22 ± 3.38	8.7 ± 3.5	>0.05
	*p*	>0.05	>0.05	
*Discrepancy*	START	10.88 ± 3.11	11.16 ± 3	>0.05
	END	6.88 ± 4.11	14.16 ± 3.2	<0.001
	*p*	<0.01	>0.05	
*Inhibition*	START	12.07 ± 2.69	11.84 ± 2.01	>0.05
	END	7.07 ± 1.97	10.84 ± 2.41	<0.001
	*p*	<0.001	>0.05	
*Tentative*	START	11.51 ± 3.13	11.46 ± 3.3	>0.05
	END	7.51 ± 2.73	16.46 ± 1.3	<0.001
	*p*	<0.001	<0.05	
*Certainty*	START	4.76 ± 1.89	5.44 ± 2.25	>0.05
	END	2.8 ± 1.9	8.74 ± 2.87	<0.001
	*p*	<0.05	<0.05	
*Sensory and perceptual processes*	START	33.36 ± 9.41	31.68 ± 10.56	>0.05
	END	40.36 ± 7.51	26.68 ± 8.56	<0.001
	*p*	<0.01	<0.01	
*Social processes*	START	21.51 ± 8.2	20.3 ± 8.27	>0.05
	END	24.12 ± 3.2	13.3 ± 4.14	<0.001
	*p*	<0.05	<0.01	
*Past tense verb*	START	14.47 ± 3.09	15.42 ± 2.92	>0.05
	END	9.47 ± 1.09	20.42 ± 3.32	<0.001
	*p*	<0.01	<0.01	
*Present tense verbs*	START	15.22 ± 3.47	14.64 ± 3.49	>0.05
	END	17.22 ± 2.17	13.64 ± 2.16	<0.01
	*p*	<0.05	<0.05	
*Future tense verb*	START	11.23 ± 3.06	10.58 ± 3	>0.05
	END	8.23 ± 2.06	13.58 ± 1.7	<0.01
	*p*	<0.01	<0.05	
*Inclusive*	START	5.28 ± 1.67	5.08 ± 1.58	>0.05
	END	9.28 ± 0.67	4.08 ± 0.86	<0.001
	*p*	<0.001	<0.01	
*Exclusive*	START	6.98 ± 1.64	7.2 ± 0.99	>0.05
	END	4.98 ± 0.94	9.2 ± 1.97	<0.001
	*p*	<0.01	<0.05	
*Motion*	START	14.81 ± 2.65	13.88 ± 2.9	>0.05
	END	17.81 ± 1.65	9.88 ± 2.9	<0.01
	*p*	<0.05	>0.05	
*Leisure activity*	START	7.2 ± 1.76	7.06 ± 1.88	>0.05
	END	14.2 ± 0.96	6.06 ± 1.85	<0.001
	*p*	<0.001	>0.05	
*Money and financial issues*	START	17.83 ± 3.72	17.76 ± 3.23	>0.05
	END	15.83 ± 4.02	22.76 ± 1.73	<0.01
	*p*	>0.05	<0.05	
*Body symptoms*	START	25.02 ± 3.89	24.98 ± 3.67	>0.05
	END	17.02 ± 2.89	21.98 ± 4.29	<0.05
	*p*	<0.01	>0.05	
*Body functions*	START	5.37 ± 2.55	5.62 ± 2.46	>0.05
	END	5.64 ± 2.92	5.42 ± 2.41	>0.05
	*p*	>0.05	>0.05	

**Table A2:** Comparison of the language analysis according to the LIWC items between RELAXATION RESPONSE group and RELAXATION RESPONSE HEALTHY CONTROLS. The *p* describing the Intra-group comparison is under, the *p* describing the inter-group comparison is on the right.

		RELAXATION RESPONSE	RELAXATION RESPONSE HEALTHY CONTROLS	*p*
*Word count*	START	344.69 ± 51.44	337.63 ± 46.15	>0.05
	END	244.69 ± 34.11	187.63 ± 41.26	<0.01
	*p*	<0.01	<0.001	
*Words per sentence*	START	22.81 ± 5.71	21.43 ± 7.52	>0.05
	END	13.54 ± 3.62	13.43 ± 2.64	>0.05
	*p*	<0.01	<0.001	
*Sentences ending with ?*	START	1.82 ± 1.07	1.95 ± 0.92	>0.05
	END	0.69 ± 0.72	0.68 ± 0.68	>0.05
	*p*	<0.01	<0.001	
*% words longer than 6 letters*	START	18.6 ± 3.62	17.62 ± 3.5	>0.05
	END	10.6 ± 1.74	8.62 ± 4.3	>0.05
	*p*	<0.001	<0.01	
*Total pronouns*	START	70.15 ± 14.04	69.64 ± 13.83	>0.05
	END	64.82 ± 14.69	69.27 ± 13.73	>0.05
	*p*	>0.05	>0.05	
*1st person singular*	START	10.15 ± 2.83	10.4 ± 2.69	>0.05
	END	5.15 ± 1.97	6.4 ± 2.28	>0.05
	*p*	<0.001	<0.01	
*1st person plural*	START	6.88 ± 3.54	6.76 ± 2.47	>0.05
	END	9.88 ± 2.44	9.76 ± 1.87	>0.05
	*p*	<0.05	<0.05	
*Total first person*	START	17.02 ± 3.62	17.16 ± 3.8	>0.05
	END	14.03 ± 2.51	16.16 ± 4.7	<0.05
	*p*	<0.05	>0.05	
*Total second person*	START	10.6 ± 2.49	10.46 ± 2.89	>0.05
	END	13.6 ± 1.18	13.86 ± 1.96	>0.05
	*p*	<0.01	<0.01	
*Total third person*	START	8.07 ± 1.97	7.67 ± 1.5	>0.05
	END	8.04 ± 1.84	8.38 ± 1.94	>0.05
	*p*	>0.05	>0.05	
*Negation*	START	16.08 ± 3.8	16.24 ± 2.69	>0.05
	END	11.08 ± 2.6	10.24 ± 3.08	>0.05
	*p*	<0.001	<0.01	
*Assents*	START	6.05 ± 1.95	5.97 ± 1.84	>0.05
	END	5.97 ± 1.89	6.02 ± 1.76	>0.05
	*p*	>0.05	>0.05	
*Articles*	START	11.74 ± 3.16	12.34 ± 3.17	>0.05
	END	8.74 ± 3.25	8.34 ± 2.34	>0.05
	*p*	<0.01	<0.01	
*Prepositions*	START	18.93 ± 4.62	18.54 ± 4.08	>0.05
	END	20.93 ± 4.12	20.54 ± 5.11	>0.05
	*p*	>0.05	>0.05	
*Numbers*	START	11.87 ± 2.31	11.94 ± 2.35	>0.05
	END	12.26 ± 2.34	12 ± 2.5	>0.05
	*p*	>0.05	>0.05	
*Affective/emotional processes*	START	24.07 ± 6.79	24.92 ± 6.46	>0.05
	END	31.07 ± 7.27	32.92 ± 5.44	>0.05
	*p*	<0.01	<0.01	
*Positive emotions*	START	17.61 ± 4.57	17.94 ± 4.56	>0.05
	END	22.61 ± 3.67	23.94 ± 2.16	>0.05
	*p*	<0.01	<0.01	
*Optimism and energy*	START	27.55 ± 5.19	27.66 ± 5.34	>0.05
	END	32.55 ± 4.68	33.66 ± 4.12	>0.05
	*p*	<0.05	<0.05	
*Negative emotions*	START	39.83 ± 9.42	27.62 ± 7.72	<0.05
	END	31.83 ± 7.31	22.62 ± 6.53	<0.01
	*p*	<0..1	<0.01	
*Anxiety or fear*	START	52.57 ± 11.39	31.84 ± 9.53	<0.001
	END	37.57 ± 9.29	21.84 ± 7.95	<0.01
	*p*	<0.001	<0.01	
*Anger*	START	25.66 ± 6.44	11.3 ± 3.2	<0.001
	END	14.66 ± 5.25	6.3 ± 2.1	<0.001
	*p*	<0.001	<0.01	
*Sadness or depression*	START	44.69 ± 9.26	19.43 ± 4.18	<0.001
	END	29.69 ± 7.76	10.43 ± 4.78	<0.001
	*p*	<0.001	<0.01	
*Cognitive processes*	START	31.42 ± 9.44	33.02 ± 7.87	>0.05
	END	24.42 ± 7.41	27.02 ± 4.11	>0.05
	*p*	<0.01	<0.05	
*Causation*	START	16.47 ± 3.67	16.64 ± 3.88	>0.05
	END	12.47 ± 2.17	11.64 ± 2.77	>0.05
	*p*	<0.01	<0.01	
*Insight*	START	9.64 ± 3.63	8.96 ± 3.07	>0.05
	END	9.22 ± 3.38	9.58 ± 3.51	>0.05
	*p*	>0.05	>0.05	
*Discrepancy*	START	10.88 ± 3.11	10.62 ± 2.9	>0.05
	END	6.88 ± 4.11	5.62 ± 2.87	>0.05
	*p*	<0.01	<0.001	
*Inhibition*	START	12.07 ± 2.69	5.31 ± 1.94	<0.001
	END	7.07 ± 1.97	2.31 ± 0.94	<0.001
	*p*	<0.001	<0.001	
*Tentative*	START	11.51 ± 3.13	6.41 ± 2.07	<0.001
	END	7.51 ± 2.73	4.41 ± 1.07	<0.01
	*p*	<0.001	<0.01	
*Certainty*	START	4.76 ± 1.89	4.9 ± 1.88	>0.05
	END	2.8 ± 1.9	2.94 ± 1.8	>0.05
	*p*	<0.05	<0.01	
*Sensory and perceptual processes*	START	33.36 ± 9.41	35.52 ± 8.8	>0.05
	END	40.36 ± 7.51	41.52 ± 6.3	>0.05
	*p*	<0.01	<0.001	
*Social processes*	START	21.51 ± 8.2	25.54 ± 8.51	<0.05
	END	24.12 ± 3.2	29.54 ± 5.32	<0.05
	*p*	<0.05	<0.05	
*Past tense verb*	START	14.47 ± 3.09	6 ± 2.23	<0.001
	END	9.47 ± 1.09	3 ± 2.03	<0.001
	*p*	<0.01	<0.01	
*Present tense verb*	START	15.22 ± 3.47	14.98 ± 3.44	>0.05
	END	17.22 ± 2.17	16.98 ± 2.48	>0.05
	*p*	<0.05	<0.01	
*Future tense verb*	START	11.23 ± 3.06	4.64 ± 1.66	<0.001
	END	8.23 ± 2.06	2.64 ± 1.97	<0.001
	*p*	<0..1	>0.05	
*Inclusive*	START	5.28 ± 1.67	10.26 ± 2.54	<0.001
	END	9.28 ± 0.67	12.26 ± 1.66	<0.001
	*p*	<0.001	<0.01	
*Exclusive*	START	6.98 ± 1.64	3.65 ± 2.19	<0.01
	END	4.98 ± 0.94	2.7 ± 2.11	<0.001
	*p*	<0.01	>0.05	
*Motion*	START	14.81 ± 2.65	14.84 ± 3.19	>0.05
	END	17.81 ± 1.65	16.84 ± 4.33	>0.05
	*p*	<0.05	>0.05	
*Leisure activity*	START	7.2 ± 1.76	11.08 ± 2.71	<0.05
	END	14.2 ± 0.96	15.08 ± 3.66	>0.05
	*p*	<0.001	<0.05	
*Money and financial issues*	START	17.83 ± 3.72	12.92 ± 2.68	<0.01
	END	15.83 ± 4.02	8.92 ± 1.03	<0.001
	*p*	>0.05	<0.01	
*Body symptoms*	START	25.02 ± 3.89	12.86 ± 2.25	<0.001
	END	17.02 ± 2.89	6.86 ± 1.74	<0.001
	*p*	<0.01	<0.001	
*Body functions*	START	5.37 ± 2.55	6.06 ± 2.65	>0.05
	END	5.64 ± 2.92	5.74 ± 2.68	>0.05
	*p*	>0.05	>0.05	
